# The Small Molecule, LLL12, Inhibits STAT3 Phosphorylation and Induces Apoptosis in Medulloblastoma and Glioblastoma Cells

**DOI:** 10.1371/journal.pone.0018820

**Published:** 2011-04-19

**Authors:** Sarah Ball, Chenglong Li, Pui-Kai Li, Jiayuh Lin

**Affiliations:** 1 Center for Childhood Cancer, The Research Institute at Nationwide Children's Hospital, Columbus, Ohio, United States of America; 2 Molecular, Cellular, and Developmental Biology Program, The Ohio State University, Columbus, Ohio, United States of America; 3 Division of Medicinal Chemistry and Pharmacognosy, College of Pharmacy, The Ohio State University, Columbus, Ohio, United States of America; Sun Yat-sen University Medical School, China

## Abstract

Tumors of the central nervous system represent a major source of cancer-related deaths, with medulloblastoma and glioblastoma being the most common malignant brain tumors in children and adults respectively. While significant advances in treatment have been made, with the 5-year survival rate for medulloblastoma at 70–80%, treating patients under 3 years of age still poses a problem due to the deleterious effects of radiation on the developing brain, and the median survival for patients with glioblastoma is only 15 months. The transcription factor, STAT3, has been found constitutively activated in a wide variety of cancers and in recent years it has become an attractive therapeutic target. We designed a non-peptide small molecule STAT3 inhibitor, LLL12, using structure-based design. LLL12 was able to inhibit STAT3 phosphorylation, decrease cell viability and induce apoptosis in medulloblastoma and glioblastoma cell lines with elevated levels of p-STAT3 (Y705). IC_50_ values for LLL12 were found to be between 1.07 µM and 5.98 µM in the five cell lines expressing phosphorylated STAT3. STAT3 target genes were found to be downregulated and a decrease in STAT3 DNA binding was observed following LLL12 treatment, indicating that LLL12 is an effective STAT3 inhibitor. LLL12 was also able to inhibit colony formation, wound healing and decreased IL-6 and LIF secretion. Our results suggest that LLL12 is a potent STAT3 inhibitor and that it may be a potential therapeutic treatment for medulloblastoma and glioblastoma.

## Introduction

While tumors of the central nervous system account for only a small percentage of cancer diagnoses, they represent a major source of cancer-related deaths. Almost 13,000 deaths occur annually in the US from primary malignant brain and CNS tumors [1]. The most common pediatric and adult CNS tumors are medulloblastoma and glioblastoma respectively [2]. Medulloblastoma accounts for approximately 20% of all pediatric CNS tumors, making it the most common malignant brain tumor in children [1]. Medulloblastomas are typically very radiosensitive tumors and the current standard for treating average risk patients is surgical resection followed by radiation and chemotherapy. However, in patients younger than 3 years of age, radiation is often avoided if possible due to the highly deleterious effects seen on the developing brain [3].

Glioblastoma is the most common brain tumor in adults [4] but 8–9% of cases are diagnosed in children [5]. The tumor is difficult to treat due to its invasive, aggressive and diffuse nature and the typical course of treatment is surgical resection, followed by radiation and chemotherapy [6], [7]. However, even with treatment, the median survival period is only 15 months [7]. The difficulty experienced trying to treat these tumors, combined with the highly toxic effects of radiation on the brains of young children, make alternative therapies highly desirable.

Signal transducer and activator of transcription 3 (STAT3) is a member of the STAT family of transcription factors which activates a variety of genes such as *c-myc, survivin, cox-2* and *cyclin D1* [8], [9], [10], [11]. Activation of STAT3 and its target genes can lead to cell-cycle progression, immune evasion, proangiogenesis, antiapoptotic effects, tumor invasion and metastasis [12], [13], all of which are typical characteristics of cancer [14]. Experiments have shown that constitutively active STAT3 alone is able to induce cellular transformation [15]. It is no surprise then that the constitutive activation of the STAT3 pathway has been found in a variety of cancers and is typically associated with a poorer prognosis [9], [12]. While STAT3 is critical during early embryogenesis, it is largely dispensable in the majority of adult cell types which makes it an attractive therapeutic target [16], [17], [18].

The inhibition of the STAT3 signaling pathway in cancer cells, using antisense oligonucleotides, RNA interference and dominant-negative STAT3, has been shown to cause decreased cellular growth and induce apoptosis [19], [20], [21], [22], [23]. Other indirect methods of inhibiting STAT3 have also been examined. The selective inhibition of the upstream tyrosine kinase, JAK2, can prevent the phosphorylation and activation of STAT3 and several JAK2 inhibitors, such as WP1066, SD-1029 and AG490, have been reported [24], [25], [26]. An intact SH2 domain is critical for STAT3 activation which makes it another reasonable target to disrupt STAT3 signaling [27]. Peptide-based SH2 inhibitors have been created, however they have low *in vivo* stability, poor cell permeability and the potential for immunogenicity [28], [29]. In order to overcome the shortcomings of peptide-based inhibitors, several non-peptide small molecular SH2 inhibitors including Stattic, STA-21 and S3I-201, have recently been reported [27], [30], [31].

Using a structure based computer design our collaborators designed a non-peptide small molecule, termed LLL12 (Figure S1). LLL12 was shown to bind directly to the phosphoryl tyrosine 705 (pY705) binding site of the STAT3 monomer on computer models with docking simulation. Here we show that LLL12 inhibits STAT3 phosphorylation, decreases cellular viability, downregulates STAT3 target gene and induces apoptosis in medulloblastoma and glioblastoma cell lines.

## Materials and Methods

### Cell Culture

The medulloblastoma cell lines (Daoy, UW426, UW288-1, D341 and D283) were provided by Dr. Corey Raffel (The Research Institute at Nationwide Children's Hospital). The glioblastoma cell lines U373 and U87Δ were provided by Dr. Sean Lawler (The Ohio State University). The WI-38 (normal human lung fibroblasts) and U87 (glioblastoma) cell lines were purchased from American Type Culture Collection. HH (human hepatocytes) were purchased from ScienCell and maintained in Hepatocyte Medium (ScienCell, #5201) supplemented with hepatocyte supplement, 5% FBS and 1% streptomycin/penicillin solution. All other cells were maintained in 1X Dulbecco's Modification of Eagle's Medium (DMEM) with 4.5 g/L, L-glutamine and sodium pyruvate (Mediatech, #10 013 CV) supplemented with 10% fetal bovine serum (FBS) (Sigma, #F1051), and 1% Penicillin/Streptomycin (P/S) (Sigma,#P0781) in incubators set at 37°C and aired with 5% CO_2_.

### Synthesis of LLL12

LLL12 was synthesized in the laboratory of Dr. Pui-Kai Li as previously described [32].

### MTT Assay

Cells were seeded in 96-well plates in triplicate at a density of 3,000 cells per well and given 24 hours to adhere. Cells were then treated with varying concentrations of the inhibitors in the presence of 10% FBS. The cells were incubated for 72 hours at 37°C. 25 µl of MTT dye (Sigma, #M5655) was added to each sample and incubated for 3.5 hours. After this, 100 µl of N,N-dimethylformamide (Sigma, #D4551) solubilization solution was added to each well. The absorbance at 450 nm was read the following day. Half-Maximal inhibitory concentrations (IC_50_) were determined using Sigma Plot 9.0 software (Systat Software Inc.).

### CyQuant NF Cell Proliferation Assay

Cells were seeded in white, clear bottom 96-well plates in triplicate at a density of 5,000 cells per well and allowed to adhere for 24 hours. Cells were then treated with varying concentrations of LLL12 in the presence of 10% FBS and incubated at 37°C for 72 hours. The medium was then removed from the cells and 100 mL of 1X dye binding solution was added and plates were incubated for one hour at 37°C. The fluorescence was then measured with excitation at 485 nm and emission detection at 530 nm.

### Western Blot Analysis

LLL12 and IL-6 (Invitrogen, #PHC0061) were both dissolved in sterile dimethyl sulfoxide (DMSO) to make stock solutions of 20 mM and 10 ng/µL respectively. The cells were grown to semi-confluency and then treated with LLL12 for either 6 or 24 hours. For the IL-6 experiments, cells were pretreated with LLL12 for 2 hours and then treated with IL-6 for 30 minutes before being harvested. For the IFN-γ (Cell Signaling Tech., #8901) and LIF (Invitrogen, #PHC9464) experiments, cells were pretreated with LLL12 for 2 hours and then treated with either IFN-γ or LIF for 24 hours. For western blots, 30 µg of total cell lysates were resolved by SDS polyacrylamide gel electrophoresis (PAGE) and transferred to PVDF membrane (GE Healthcare, #45-000-931). These membranes were then blotted with phospho-specific STAT3 antibody [Tyrosine 705] (#9131), phospho-independent STAT3 antibody (#9132), cleaved caspase-3 [Asp175] antibody (#9661), phospho-specific AKT [Serine 473] (#9271), phospho-specific ERK [Threonine 202/Tyrosine 204] (#9101) and GAPDH antibody (#2118). All antibodies were purchased from Cell Signaling Tech. Membranes were analyzed with enhanced chemiluminescence Plus reagents (GE Healthcare, #RPN5781) and scanned with a Storm Scanner (Amersham Pharmacia Biotech Inc.). Integrated densities of the bands in the western blots were measured using Image J software (NIH). Densities were individually normalized to GAPDH in each cell line and the relative levels of p-STAT3, STAT3, cleaved caspase-3, p-AKT and p-ERK were compared to the DMSO control, which was set as 1.0.

### Reverse-transcriptase PCR

Following 24 hours of treatment with LLL12, RNA was collected from cells using a RNeasy Kit (Qiagen, #74104). cDNA was generated from 500 ng of sample RNA using Omniscript RT (Qiagen, #205111). Subsequently, 2 µl of cDNA was used for PCR. PCR amplifications were performed as follows: 5 min at 94°C followed by 25 cycles of [30 sec at 94°C, 30 sec at 55°C, 30 sec at 72°C] and a final extension at 72°C for 5 min. The PCR products were then run on 2% agarose gels, stained with ethidium bromide and visualized under UV light.

### Immunofluorescence

Cells were seeded on coverslips and grown until 80% confluent. After either 6 or 24 hours of treatment with LLL12, cells were fixed with ice-cold methanol for 30 minutes, washed and blocked in 5% normal goat serum (Jackson ImmunoResearch Laboratories, #005-000-121) in PBS for 1 hour at room temperature. The coverslips were then incubated overnight at 4°C in a 1:100 dilution of cleaved caspase-3 (Asp175) antibody (Cell Signaling Tech., #9661), washed and then incubated at room temperature in a 1:1000 dilution of Alexa Fluor 594 conjugated goat anti-rabbit IgG antibody (Invitrogen, #A-11037). Fluorescence staining was examined using a Leica MZ 16FA inverted microscope (Leica Microsystems, Bannockburn, IL) with a 7.4 slider digital camera (Diagnostic Instruments Inc.).

### Colony Formation

Cells were grown to semi-confluency in 10 cm plates and then treated for either 6 hours or 24 hours. Cells were then trypsinized, stained with trypan blue and counted. A low number of cells (3,000 for UW288-1, 1,000 for U87 and 500 for U87Δ) were then seeded on 15cm plates in duplicate and allowed to grow for two weeks. Cells were then fixed in methanol for 30 minutes and stained with 1% crystal violet dye.

### Cell Death ELISA

Cells were seeded in 96 well plates at a density of 10^4^ cells per well and allowed to adhere overnight. They were then treated with 5 µM of LLL12 for either 18 or 24 hours. Cells were lysed directly in the plate and 20 µl of lysate was transferred to a new plate and the level of apoptosis was measured using the Cell Death Detection ELISA^PLUS^ assay following the manufacturer's protocol (Roche, #11774425001).

### DNA Binding Assay

Cells were grown to semi-confluency in 10 cm plates and then treated with varying concentrations of LLL12 for either 18 or 24 hours. Cells were collected and the nuclear fraction was extracted using a Nuclear Extract Kit (Active Motif, #40010) according to the manufacturer's protocol. 20 µg of nuclear extract was used to analyze STAT3 activation using the TransAM STAT3 Activation Assay (Active Motif, #45196) following the manufacturer's protocol. Samples were assayed in triplicate and the error bars represent one standard deviation.

### Wound Healing Assay

Cells were seeded in 6 well plates and allowed to grow until confluent. They were then treated with varying amounts of LLL12 and the tip of a 1–10 µl disposable pipette tip was used to create a wound by dragging it across the surface of the plate and dislodging a line of cells. Cells were allowed to grow for 48 hours and images were captured using a Leica MZ 16FA inverted microscope (Leica Microsystems) with a 7.4 slider digital camera (Diagnostic Instruments Inc.).

### ELISA

To measure baseline levels of IL-6, cells were grown in 6 well plates in DMEM supplemented with 10% FBS until ∼70% confluent. The media was then replaced and cells were allowed to continue growing for 48 hours before the media was collected, filter-sterilized and stored at −80°C. To examine the effect of LLL12 on IL-6 secretion, cells were grown in 6 well plates until ∼70% confluent and then the media was replaced and cells were treated with either DMSO (control) or LLL12. Media was collected at 8, 16 and 24 hours, filter-sterilized and stored at −80°C. IL-6 secretion was quantified using an IL-6 ELISA kit (PeproTech, #900-K16) according to the manufacturer's protocol. The same samples were also used to measure LIF secretion (RayBiotech Inc., #ELH-LIF-001). Samples were assayed in triplicate and error bars represent one standard deviation.

## Results

### LLL12 inhibits cellular viability/proliferation in human medulloblastoma and glioblastoma cell lines

**Figure 1:**
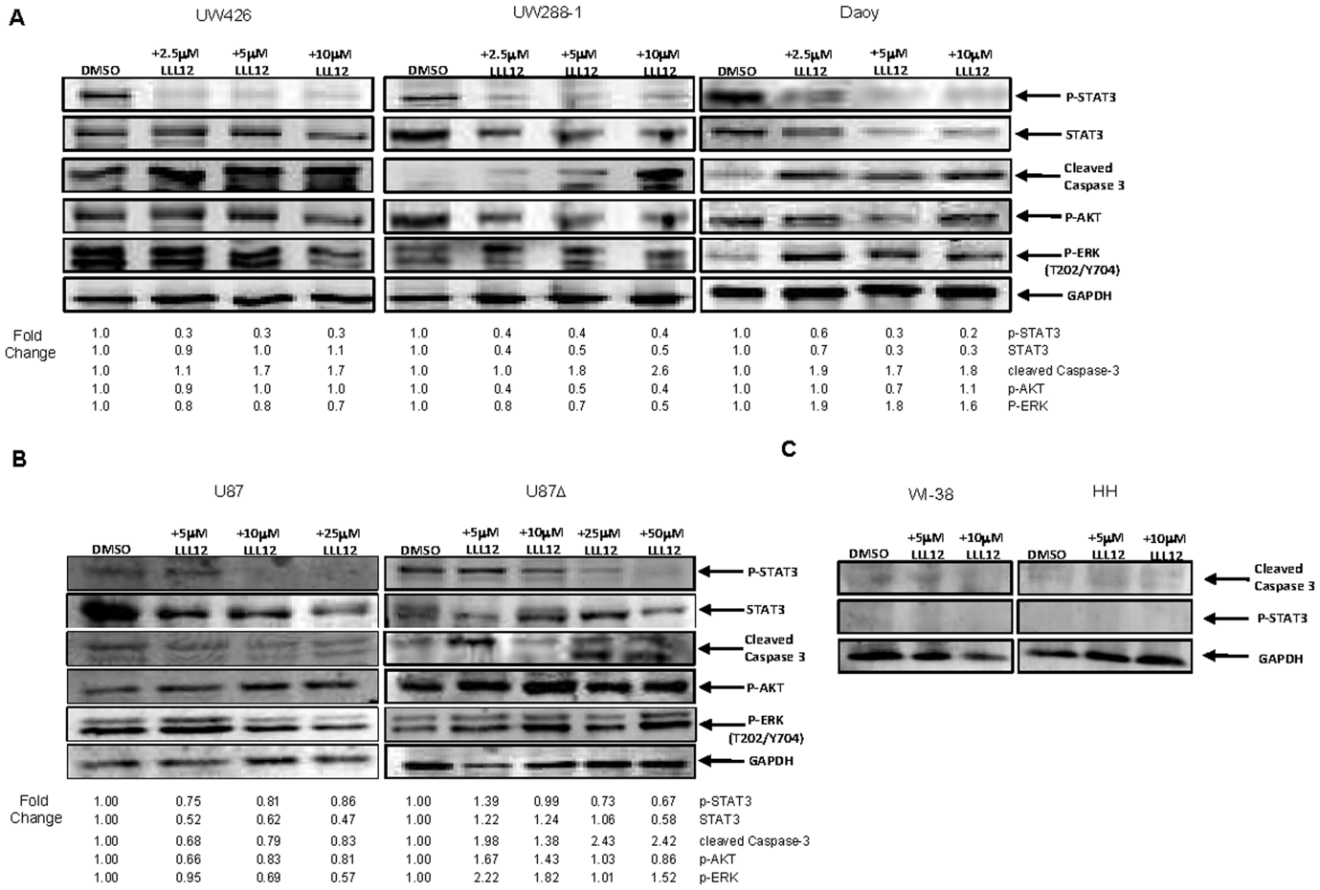
Western blot analysis of cells treated with LLL12. (A) Medulloblastoma cell lines and (B) glioblastoma cell lines which express constitutively active STAT3 exhibit a decrease in p-STAT3 (Y705) following treatment with LLL12 for 6 and 24 hours respectively. No effect is seen on other kinases such as p-AKTand pERK. Apoptosis is indicated by the cleavage of caspase 3. (C) Normal human lung fibroblasts (WI-38) and human hepatocytes (HH) that do not express p-STAT3, did not show an induction of cleaved caspase-3.

Cell Viability assays were run on several human medulloblastoma and glioblastoma cells lines which express elevated levels of phosphorylated STAT3 (Figure 1A and 1B) in order to assess LLL12's inhibitory effects. After 72 hours of treatment, a dose-dependent inhibition of cellular viability was seen. IC_50_ values were calculated for LLL12 and other previously reported inhibitors (Table 1); LLL3 [33] and S3I-201 [31], both STAT3 inhibitors, and AG490 [34], a JAK2 inhibitor. LLL12 was found to be much more potent than the other inhibitors tested in the inhibition of cell viability and proliferation. Cellular viability was confirmed using a CyQuant NF assay, which measures cellular content via fluorescent dye binding (data not shown). The calculated IC_50_ values for LLL12 were very similar to those determined by MTT, ranging from 0.7 µM to 3.68 µM.

**Table 1:** IC_50_ values for medulloblastoma and glioblastoma cell lines.

	LLL12	LLL3	AG490	S3I-201
Daoy	2.79	82.81	20.9	>100
UW426	4.03	17.45	>100	>100
UW288-1	1.07	22.0	>100	>100
U87	1.99	12.9	>100	>100
U87Δ	5.98	14.8	>100	>100

The half-maximal inhibitory concentrations (IC_50_) calculated for LLL12 and other STAT3/JAK2 inhibitors (µM) in medulloblastoma and glioblastoma cell lines. Cellular proliferation was measured using a MTT Assay following 72 hours of treatment.

### LLL12 inhibits STAT3 phosphorylation and induces apoptosis in human medulloblastoma and glioblastoma cell lines

**Figure 2:**
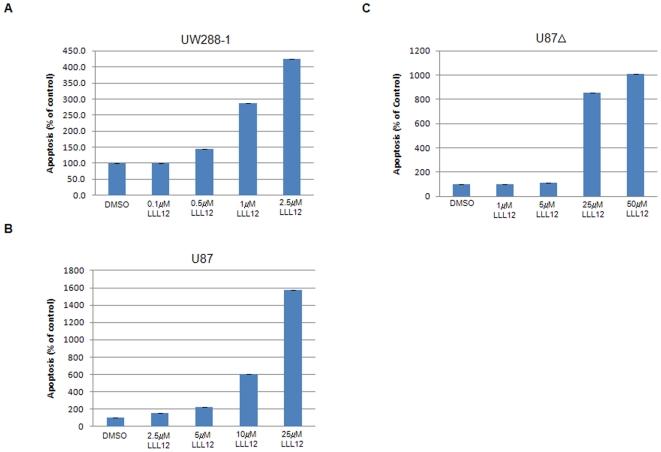
Cell Death ELISA analysis following LLL12 treatment. (A) UW288-1, (B) U87, and (C) U87Δ exhibit a dose dependent increase in apoptosis following treatment with LLL12 for either 18 hours (UW288-1) or 24 hours (U87 and U87Δ).

Several medulloblastoma and glioblastoma cell lines which overexpress phosphorylated STAT3 (Daoy, UW426, UW288-1, U87 and U87Δ) were used to evaluate the effects of LLL12 on STAT3 phosphorylation and the induction of apoptosis. LLL12 inhibited the phosphorylation of STAT3 at tyrosine residue 705 (Y705) in all cell lines tested (Figure 1A and 1B). LLL12 did not inhibit the phosphorylation of other kinases, such as ERK 1/2 and AKT, indicating that LLL12 is specific for STAT3. LLL12 was also able to induce apoptosis as evidenced by the cleavage of caspase-3 (Figure 1A and 1B) which is consistent with the inhibition of p-STAT3 (Y705) seen. LLL12 did not induce apoptosis in normal human hepatocyte and lung fibroblast cell lines, indicating that its toxicity is confined to cancer cells which express p-STAT3 (Figure 1C). Using a Cell Death Detection ELISA, which measures cytoplasmic histone-associated-DNA-fragments, we saw a dose-dependent increase in apoptosis in UW288-1, U87 and U87Δ cell lines after 18 and 24 hours of treatment with LLL12 respectively (Figure 2A–2C). Cell death through apoptosis was additionally confirmed in UW288-1, U87 and U87Δ cell lines using immunofluorescence to look for the cleaved form of caspase 3 (Figure S2).

### LLL12 inhibits the transcription of downstream STAT3 target genes

**Figure 3:**
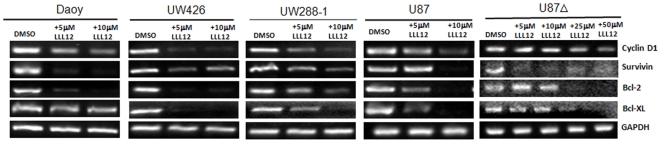
RT-PCR Analysis of STAT3 target genes. LLL12 treatment was able to downregulate the expression of STAT3 target genes: Cyclin D1, Survivin, Bcl-2 and Bcl-xL following 24 hours of treatment.

As previously mentioned, STAT3 activates the transcription of a variety of genes responsible for cell cycle regulation, anti-apoptotic effects and other hallmarks of cancer. Using Reverse Transcriptase PCR, we examined LLL12's effect on the activation of these genes after 24 hours of treatment. We found that LLL12 inhibited the transcription of the STAT3 downstream target genes, cyclin D1, survivin, Bcl-2, and Bcl-xL in medulloblastoma and glioblastoma cell lines (Figure 3).

### LLL12 is specific for STAT3 and does not inhibit STAT1

Two cell lines which express lower levels of p-STAT3 (Y705), D283 (medulloblastoma) and U373 (glioblastoma), were used to assess LLL12's specificity. Cells were treated with IFN-γ in order to stimulate the phosphorylation of STAT1 following pretreatment with LLL12. LLL12 was not able to block the activation of STAT1, indicating that it is specific for STAT3 (Figure S3).

### LLL12 inhibits STAT3 DNA binding activity

**Figure 4:**
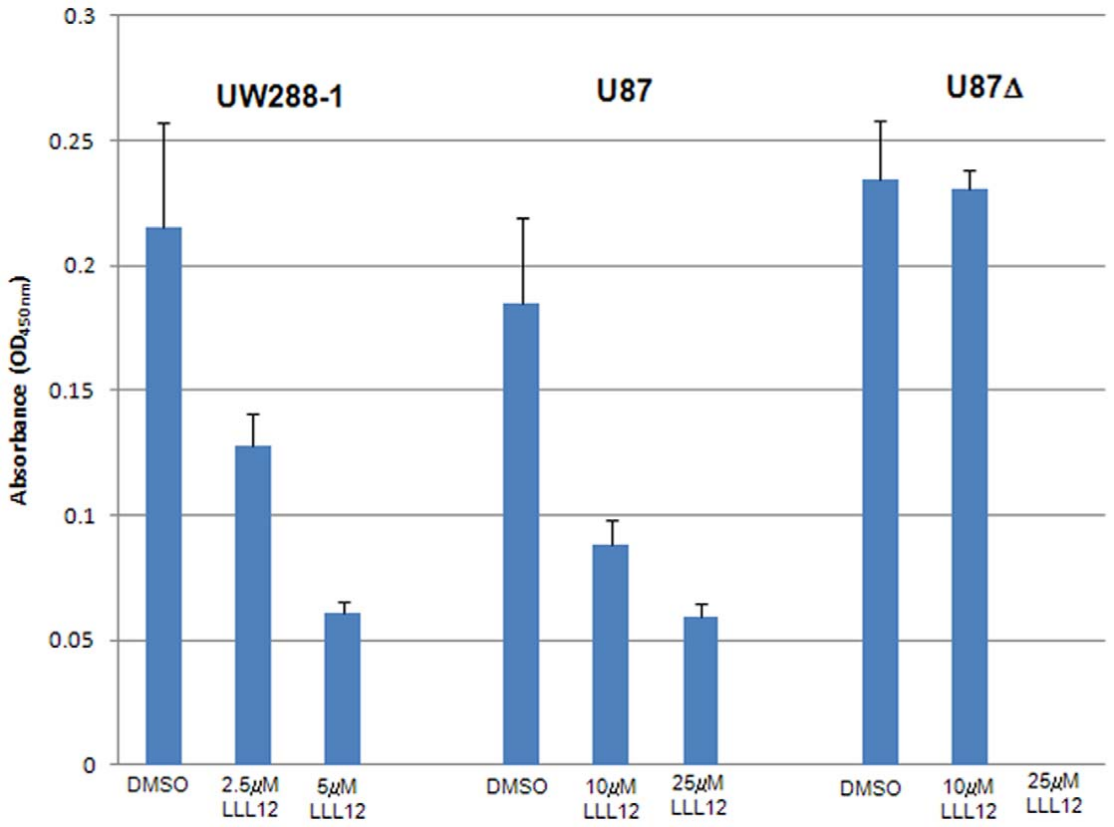
STAT3 DNA Binding Assay. Cells showed a dose-dependent decrease in STAT3 activation following treatment with LLL12 for either 18 hours (UW288-1) or 24 hours (u87 and U87Δ), indicating that LLL12 is able to effectively inhibit STAT3's ability to bind DNA.

We examined LLL12's effect on DNA binding activity in order to assess the drug's ability to inhibit STAT3 signaling. Using an ELISA-based assay, we measured the DNA binding ability of STAT3 in the cell lines UW288-1, U87 and U87Δ. We found a dose-dependent decrease in STAT3 binding (Figure 4). This method was previously utilized in our lab to examine LLL12's effect on STAT1 DNA binding and we found no inhibition of STAT1 DNA binding activity, indicating once again that LLL12 is a specific inhibitor of STAT3 [32].

### Wound Healing and colony formation are inhibited in the presence of LLL12

**Figure 5:**
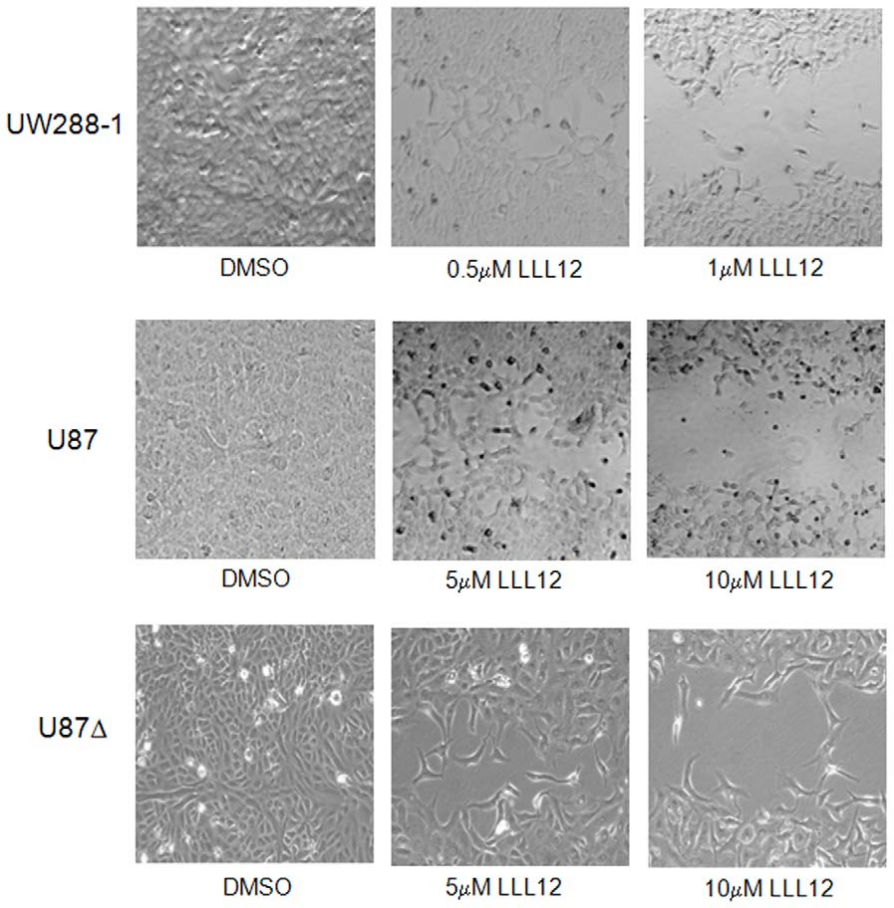
Wound Healing Assay. Cells showed a dose-dependent decrease in their ability to migrate and heal the created wound following treatment with LLL12.

Many cellular processes, such as proliferation, angiogenesis, invasion and migration are common to both wound healing and cancer [35] and these similarities have given rise to the concept that tumors are “wounds that do not heal” [36]. In order to assess LLL12's ability to inhibit wound healing, a wound healing assay was performed on UW288-1, U87 and U87Δ cells. After the creation of a wound, cells were treated with varying concentrations of LLL12 and allowed 48 hours to proliferate and migrate into the wound. Treatment with LLL12 resulted in a decreased ability for cells to migrate and heal the created wound (Figure 5).

**Figure 6:**
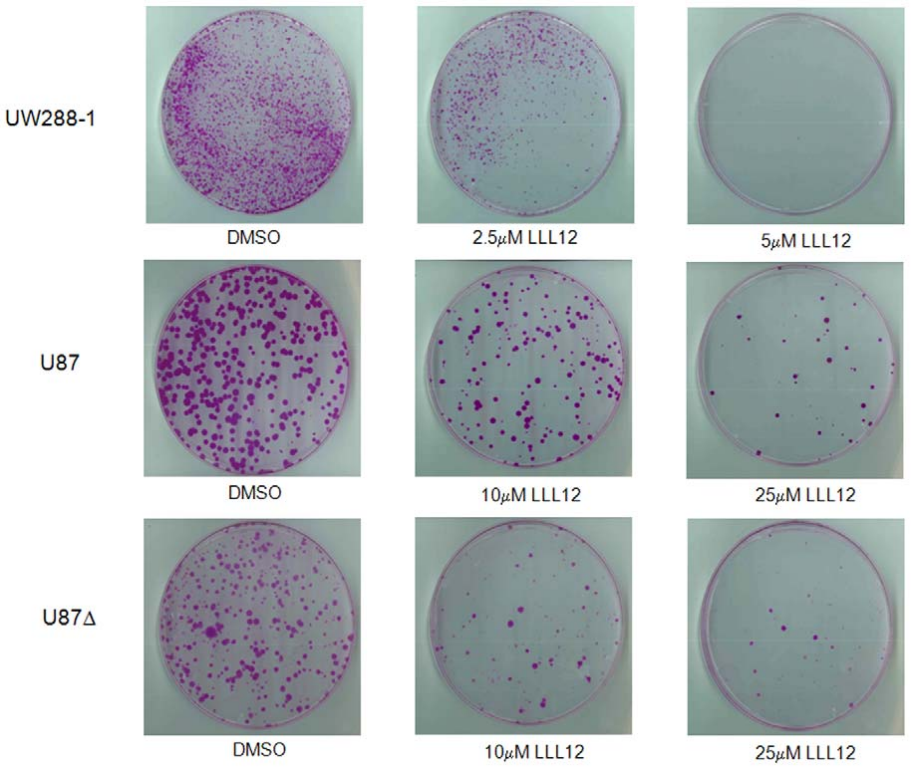
Colony Formation Assay. Medulloblastoma and glioblastoma cell lines showed a decreased ability to form colonies following treatment with LLL12 for either 6 hours (UW288-1) or 24 hours (U87 and U87Δ).

We also examined the ability of the cells to recover after treatment with LLL12 by performing a colony formation assay. The cell lines UW288-1, U87 and U87Δ were treated with LLL12 for 4 and 24 hours respectively and then the same number of living cells was reseeded at very low cell densities and allowed to grow for 2 weeks. Cells were then fixed and stained and the plates were scanned. The cancer cells showed a decreased ability to recover and form colonies following treatment with LLL12 (Figure 6).

### LLL12 is able to inhibit the secretion of IL-6 and LIF in Medulloblastoma and Glioblastoma cell lines

**Figure 7:**
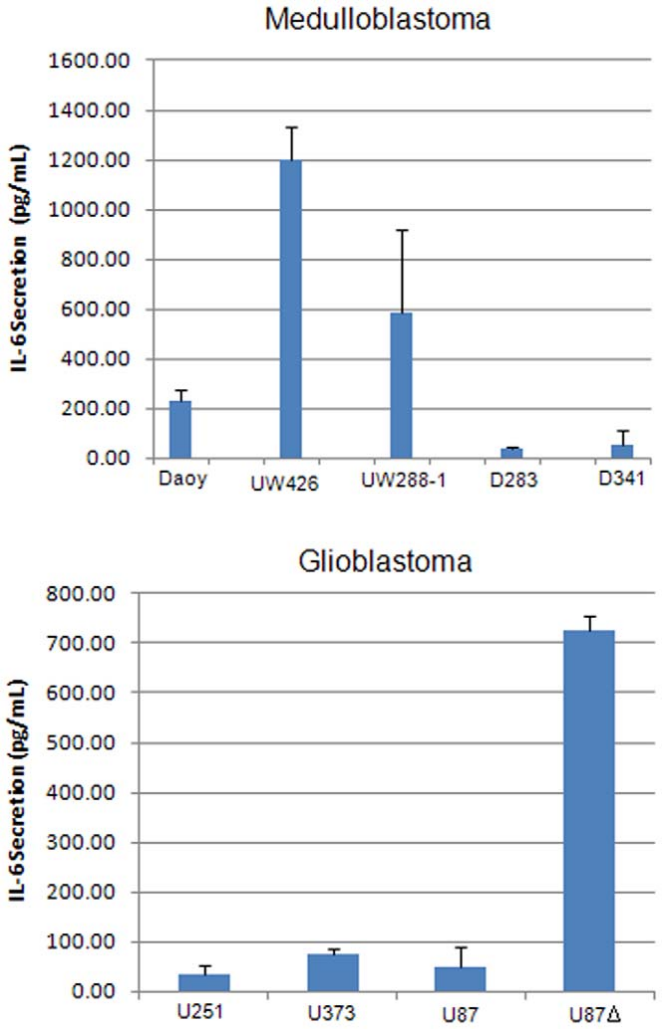
IL-6 secretion in medulloblastoma and glioblastoma cell lines. IL-6 secretion was measured by ELISA after 48 hours. The cell lines UW288-1, UW426 and U87Δ secreted elevated levels of IL-6.

**Figure 8:**
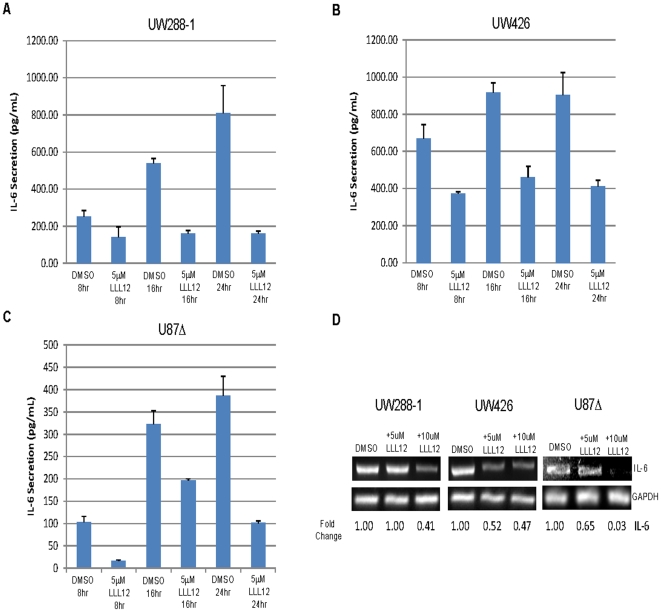
LLL12 inhibition of IL-6 secretion. (A–C) Cells were treated with LLL12 for 8, 16 and 24 hours and IL-6 levels were measured using an ELISA. IL-6 secretion was reduced at all three time points. (D) LLL12 inhibits the expression of IL-6 mRNA.

**Figure 9:**
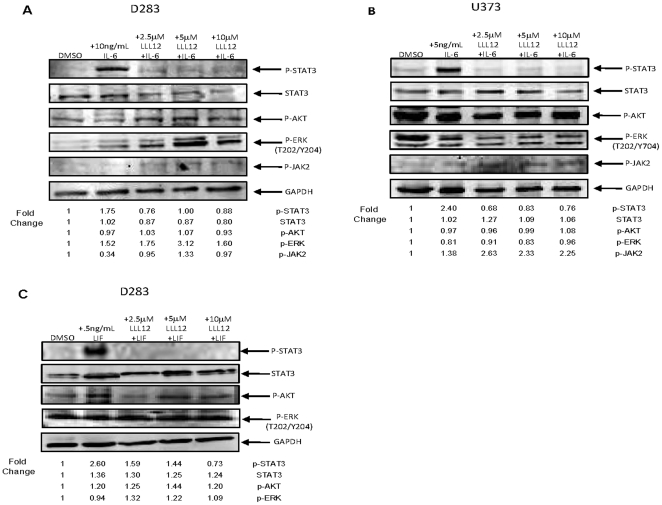
LLL12 inhibits IL-6 and LIF induced STAT3 phosphorylation. (A) D283 and (B) U373 cells were pretreated with LLL12 for 2 hours and then treated with IL-6 for 30 minutes to induce p-STAT3. LLL12 was able to block STAT3 activation by IL-6 and did not have an effect of p-AKT or p-ERK. (C) D283 cells were pretreated with LLL12 for 2 hours and then treated with LIF for 24 hours to activate STAT3. LLL12 was able to inhibit the phosphorylation of STAT3 induced by LIF and did not inhibit p-AKT or p-ERK expression.

IL-6 is a pleiotropic cytokine which has been shown to be overexpressed in response to infection, injury and inflammation. Many tumor cells have been found to produce excess amounts of IL-6 or alternatively, express an IL-6 receptor which allows them to respond to IL-6 produced by the tumor microenvironment [37]. The binding of IL-6 to its receptor leads to the activation of the Janus kinase family which in turn phosphorylate STAT3 [38]. Using an ELISA, we measured the amount of IL-6 secreted by our medulloblastoma and glioblastoma cell lines. We found that UW288-1, UW426 and U87Δ secreted measurably higher levels of IL-6 than the other cell lines tested (Figure 7). Next we wanted to see whether or not LLL12 could block the secretion of IL-6 since the gene encoding it is one of STAT3's targets [39]. Cells were treated with LLL12 and their media was collected after 8, 16 and 24 hours for analysis. LLL12 was able to inhibit the secretion of IL-6 in UW288-1, U87 and U87Δ cells as early as 8 hours after treatment and the decrease was still seen at 24 hours post treatment (Figure 8A–8C). We also saw a decrease in the expression of IL-6 mRNA following LLL12 treatment (Figure 8D). We also looked at LLL12's ability to inhibit the induction of p-STAT3 following IL-6 treatment in cell lines that do not express activated STAT3. Cells were pretreated with LLL12 for 2 hours and then IL-6 was added for 30 minutes. We found that LLL12 was able to completely block the phosphorylation of STAT3 caused by IL-6 treatment (Figure 9A and 9B).

We also wanted to examine the secretion of leukemia inhibitory factor (LIF) because it is an IL-6 family member [40] that has been shown to be constitutively expressed both *in vitro* and *in* vivo in medulloblastoma cells [41]. We found elevated levels of LIF in UW288-1 and UW426 cells and treatment with LLL12 was able to inhibit LIF secretion and decrease the expression of LIF mRNA (Figure S4A–4C). We also wanted to see if LLL12 could block the activation of STAT3 by LIF. Cells were pretreated with LLL12 for 2 hours and then treated with LIF for 24 hours. LLL12 was found to be able to inhibit the phosphorylation of STAT3 caused by LIF treatment but had no effect on the phosphorylation of AKT or ERK (Figure 9C).

## Discussion

Despite several new treatment options, the prognosis for patients diagnosed with glioblastoma remains poor with a median survival of 15 months for glioblastoma patients [7] and although treatment for medulloblastoma has proven more effective, resulting in a 5-year survival of 70–80% [42], the long term effects can be severe [2]. More effective and less toxic treatment options need to be developed to increase patient survival rates and combat these devastating tumors. Our collaborators developed a non-peptide small molecule inhibitor of STAT3, LLL12, which has shown promising results in the treatment of several types of cancer [32].

We examined the effects of LLL12 on medulloblastoma and glioblastoma cell lines which express phosphorylated STAT3. We found that LLL12 is able to inhibit cell viability, decrease STAT3 target gene expression, decrease STAT3 DNA binding, inhibit wound healing and colony formation and induce apoptosis. LLL12 did not have any effect on the phosphorylation of ERK or AKT, and it did not inhibit STAT1 activation caused by IFN-γ treatment, indicating that it is a specific inhibitor of STAT3. The cytokine IL-6 has been found to be a major contributor to the tumor microenvironment and many tumors have been found to express high levels of IL-6 or an IL-6 receptor [37], [43]. We found that UW288-1, UW426 and U87Δ secrete high levels of IL-6 and that LLL12 was able to decrease IL-6 expression and secretion. This is significant because while not all tumors overexpress activated STAT3, IL-6 secreted in the microenvironment may be able to activate STAT3 and LLL12 can block that activation and inhibit the pro-tumorigenic effects of STAT3. We also examined the ability of LLL12 to inhibit STAT3 activation caused by LIF. LIF has been shown to be constitutively expressed in medulloblastoma cells [41] and we found high levels of LIF secreted in UW288-1 and UW426 cells. Treatment with LLL12 was able to block the secretion of LIF and downregulate the expression of LIF, however the exact mechanism is unclear since LIF is not a target gene of STAT3 like IL-6.

LLL12's drug-likeness characteristics were previously evaluated using parameters such as molecular weight, cell permeability, solubility, and metabolic stability and its toxicity, absorption, metabolism and excretion were also measured. LLL12 was shown to have decent drug-like properties which warrant further investigation [32].

One of the problems encountered when trying to treat brain tumors is drug delivery across the blood brain barrier (BBB). There have been many new techniques and strategies employed to disrupt the BBB or to deliver drugs across the barrier including: transient osmotic BBB disruption (BBBD), biochemical BBBD, ultrasound-mediated BBBD, implanted polymers, intra-cavitary delivery systems and convection-enhanced delivery [44] but these techniques need to be paired with novel therapies in order to truly gauge the effectiveness of new treatments. We have shown LLL12 to be an effective inhibitor of STAT3 and it has been shown to reduce to reduce tumor size *in vivo* [32] but it remains to be seen whether or not LLL12, in conjunction with available BBBD methods, can effectively cross the barrier and suppress tumor growth. Additional studies need to be done employing more advanced mouse models and BBBD techniques to further evaluate LLL12's effectiveness against glioblastoma and medulloblastoma but our early findings indicate that LLL12 merits further investigation.

## Supporting Information

Figure S1 **Chemical Structure of LLL12.**
(TIF)

Figure S2 **Immunofluorescence for cleaved caspase-3.** Cells were treated with LLL12 for either 6 (UW288-1) or 24 hours (U87 and U87Δ) and stained for cleaved caspase-3. LLL12 induced apoptosis in all cell lines as evidenced by the presence of cleaved caspase-3.
(TIF)

Figure S3 **LLL12 does not inhibit IFN-γ induced STAT1 activation.** D283 and U373 cells were pre-treated with LLL12 for 2 hours and then treated with IFN-γ for 24 hours. IFN-γ induced the phosphorylation of STAT1 but pre-treatment with LLL12 was not able to inhibit this induction, indicating it is specific for STAT3.
(TIF)

Figure S4 **LIF Secretion in medulloblastoma cell lines.** (A) ELISA analysis showed elevated levels of LIF in UW288-1 and UW426 cell lines. (B) LLL12 was able to block the secretion of LIF in UW426 cells. (C) LLL12 was able to downregulate the expression of LIF mRNA.
(TIF)
