# Supplementary figures and images for: The Small Molecule, LLL12, Inhibits STAT3 Phosphorylation and Induces Apoptosis in Medulloblastoma and Glioblastoma Cells
Source: PLoS One. 2011 Apr 19;6(4):e18820. doi: 10.1371/journal.pone.0018820 (PMC3079737; doi:10.1371/journal.pone.0018820)

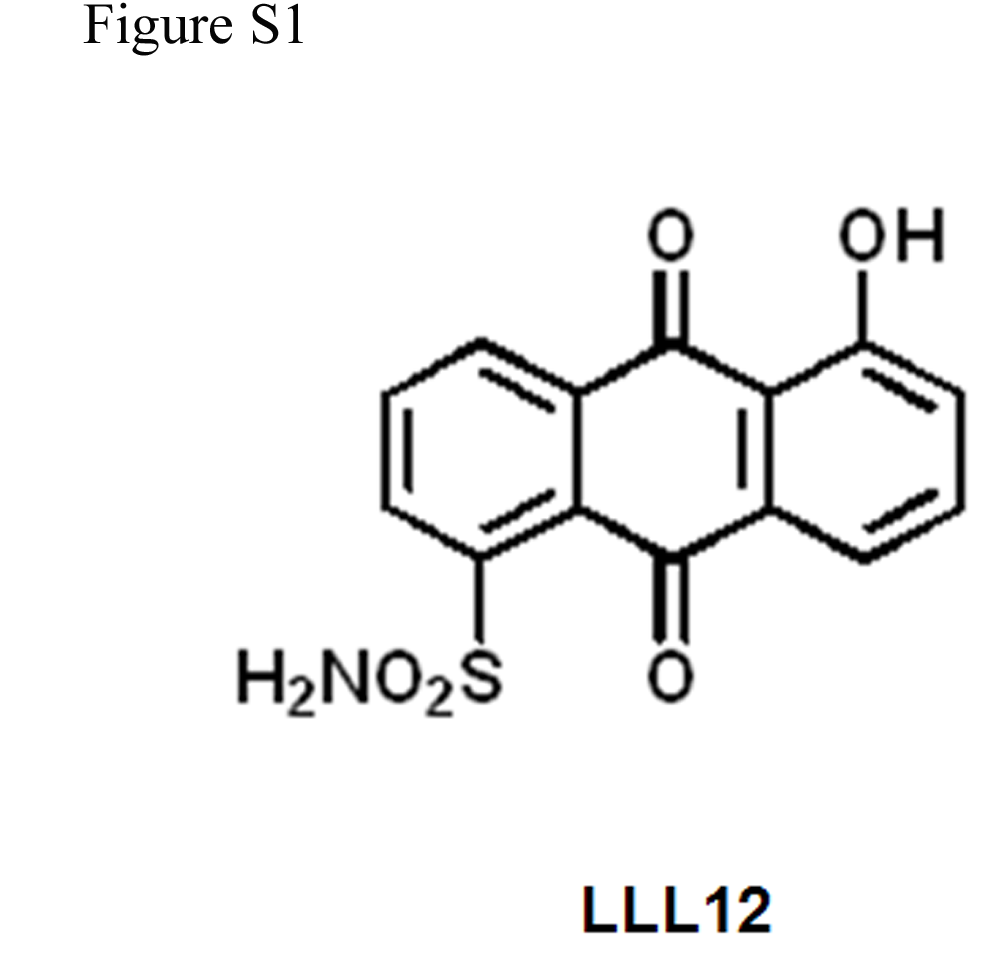

Supplement: Figure S1 — Chemical Structure of LLL12. (TIF) [file pone.0018820.s001.tif]

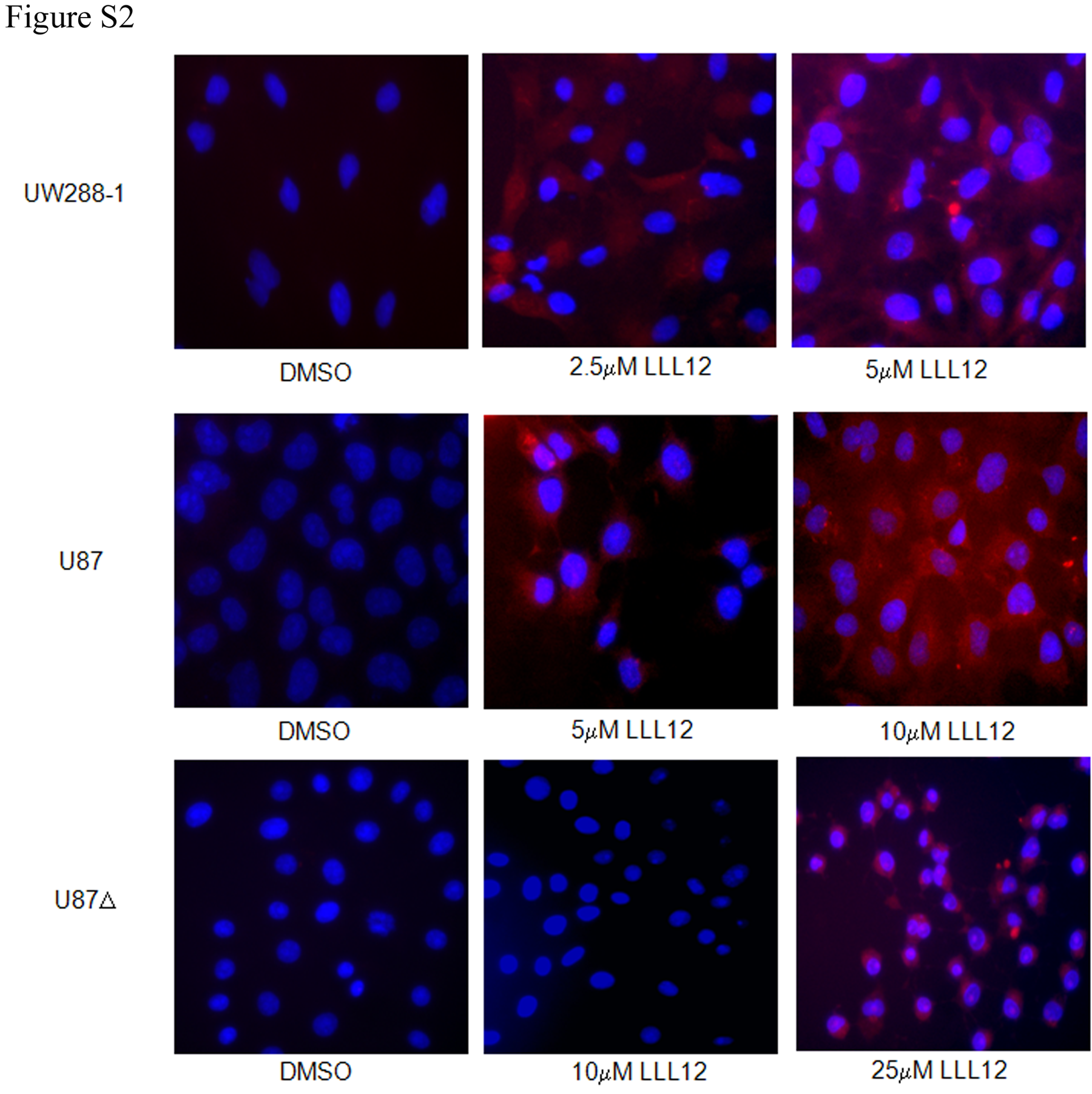

Supplement: Figure S2 — Immunofluorescence for cleaved caspase-3. Cells were treated with LLL12 for either 6 (UW288-1) or 24 hours (U87 and U87Δ) and stained for cleaved caspase-3. LLL12 induced apoptosis in all cell lines as evidenced by the presence of cleaved caspase-3. (TIF) [file pone.0018820.s002.tif]

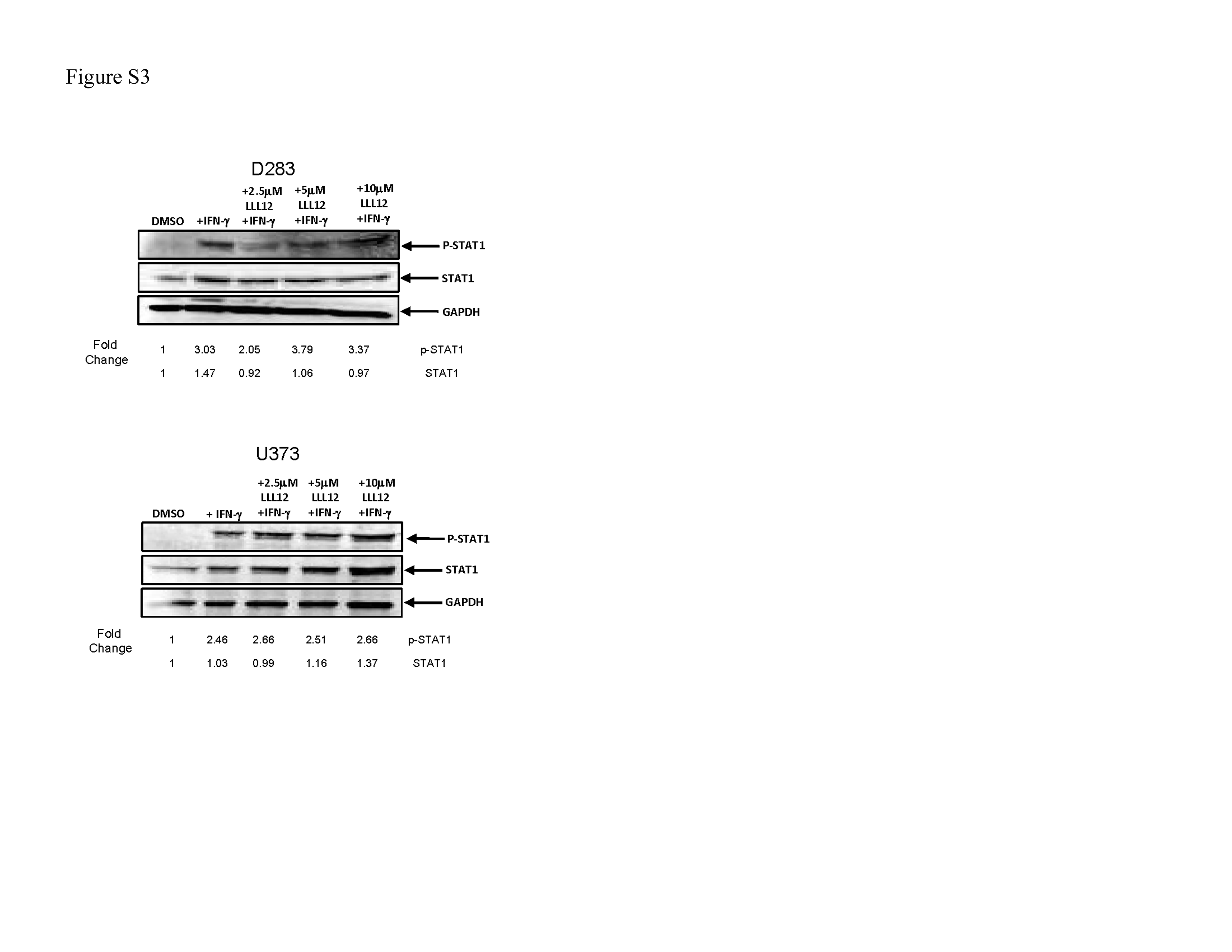

Supplement: Figure S3 — LLL12 does not inhibit IFN-γ induced STAT1 activation. D283 and U373 cells were pre-treated with LLL12 for 2 hours and then treated with IFN-γ for 24 hours. IFN-γ induced the phosphorylation of STAT1 but pre-treatment with LLL12 was not able to inhibit this induction, indicating it is specific for STAT3. (TIF) [file pone.0018820.s003.tif]

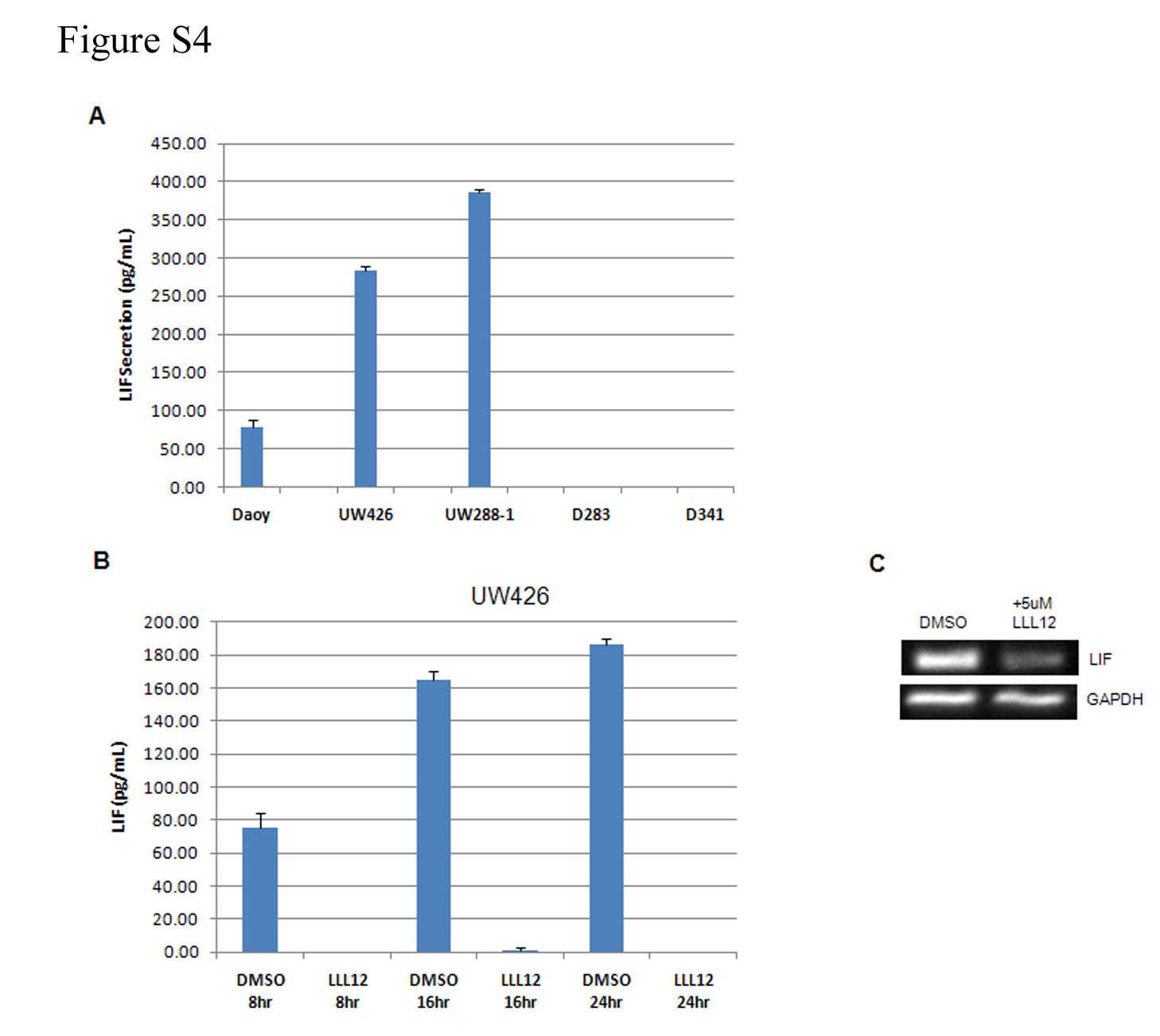

Supplement: Figure S4 — LIF Secretion in medulloblastoma cell lines. (A) ELISA analysis showed elevated levels of LIF in UW288-1 and UW426 cell lines. (B) LLL12 was able to block the secretion of LIF in UW426 cells. (C) LLL12 was able to downregulate the expression of LIF mRNA. (TIF) [file pone.0018820.s004.tif]
